# Judicial hierarchy and discursive influence

**DOI:** 10.1098/rsta.2023.0145

**Published:** 2024-04-15

**Authors:** Felix Herron, Keith Carlson, Daniel N. Rockmore, Michael A. Livermore

**Affiliations:** ^1^ Sorbonne Université, Paris 75006, France; ^2^ Dartmouth College, Hanover, NH 03755, USA; ^3^ The Santa Fe Institute, Santa Fe, NM 87501, USA; ^4^ University of Virginia, Charlottesville, VA 22902, USA

**Keywords:** law, supreme court, federal courts, topic model, dynamic topic model, document influence

## Abstract

We apply a dynamic influence model to the opinions of the US federal courts to examine the role of the US Supreme Court in influencing the direction of legal discourse in the federal courts. We propose two mechanisms for how the Court affects innovation in legal language: a selection mechanism where the Court's influence primarily derives from its discretionary jurisdiction, and an authorship mechanism in which the Court's influence derives directly from its own innovations. To test these alternative hypotheses, we develop a novel influence measure based on a dynamic topic model that separates the Court's own language innovations from those of the lower courts. Applying this measure to the US federal courts, we find that the Supreme Court primarily exercises influence through the selection mechanism, with modest additional influence attributable to the authorship mechanism.

This article is part of the theme issue ‘A complexity science approach to law and governance’.

## 1. Introduction

Changes in formal legal rules announced by courts are only one mechanism through which the law dynamically responds to its economic, political, social and cultural milieu. The subject matter of the law also shifts, as, for example, new technologies or social relations give rise to entirely different classes of legal disputes. More subtle are variations over time in how judges discuss the facts and the law in the cases before them. Such discursive shifts affect the framing and analysis of legal questions within broader subject matter categories.

An important set of questions in a hierarchically organized judicial system concerns the locus of change. Apex courts, such as the Supreme Court in the United States, authoritatively settle disputes and announce doctrine. But their role in affecting judicial discourse more broadly is more ambiguous. Litigants, lower courts and broader social, political and economic forces also play a role in determining which matters are brought to courts for adjudication, and what receives the most attention in judicial opinions [1,2].

In this paper we explore two mechanisms of this *discursive influence*: a *selection* mechanism and an *authorship* mechanism. These distinct influence mechanisms derive from the Supreme Court's two most important powers, which are the ability to take up cases for review (i.e. the *certiorari* power); and the ability to author and publish opinions. The selection mechanism is associated with the *certiorari* power, and the authorship mechanism is associated with the Supreme Court's opinions.

The selection mechanism is roughly Darwinian, which is to say that it is driven by variation and selection. Variation occurs in the lower courts, as judges introduce variants of existing discursive forms. This variation is not designed to achieve discursive influence but is rather a by-product of the standard operation of the lower courts. The Supreme Court takes on the role of the evolutionary environment: variants have an increased likelihood of reproduction when they are selected by the Court for review. The discursive variants in a lower court opinion are more ‘fit’ (i.e. more likely to ‘reproduce’ by being taken up by future courts) when the Supreme Court is more likely to select cases with those variants for review.

The authorship mechanism is operative when the Court introduces innovative language usage and lower courts take up and propagate those innovations. Lower court judges may copy the Supreme Court's language usage patterns out of deference, or due to the Supreme Court's prestige, or simply due to exposure: opinions issued by the Supreme Court are far more widely read than those of another US court. There is some degree of a Darwinian process in the authorship mechanism as well; lower courts may preferentially select some of the discursive innovations (variants) that are generated by the Supreme Court for future copying.

In this paper, we empirically test the degree to which the corpus of published judicial opinions in the United States in the period 1975–2005 is more consistent with the selection or authorship mechanism of hierarchical discursive influence. The selection mechanism would lead innovative language to be found in lower court opinions first, before being taken up by the Supreme Court and being propagated forward. The authorship mechanism would lead to the most innovative language being found in Supreme Court opinions.

Our method is based on identifying those documents that contain the most innovative language, in the sense of predicting future shifts in legal discourse. Building on prior work on topic models [3], dynamic topic modelling [4] and influence modelling [5,6], we operationalize this notion of innovation as changes in the distribution of the words associated with a given subject over time. This work extends the already substantial body of scholarship applying topic-model-based tools to the law [7,8].

With this model we find that the Supreme Court's discursive influence is substantially attributable to selection, with authorship playing a secondary role. Cases that are selected by the Supreme Court for review have substantially more innovative language than average appellate court cases. Furthermore, among Supreme Court cases, those that were taken up under the *certiorari* mechanism have discursive influence while non-*certiorari* cases do not. We find some evidence that authorship plays some role in the Supreme Court's discursive influence by comparing models in which Supreme Court opinions are included with other models that excluded those opinions. Overall, we find evidence that both the selection and authorship mechanisms help explain the Supreme Court's discursive influence, with the former dominating the latter.

## 2. Method

### (a) Mechanisms of discursive influence

Theorists have long conceptualized the law as a set of formal rules that could even, in principle, be computed algorithmically [9–11]. We refer to this set of theories as the *formal school*. An alternative perspective, associated with the law and literature movement and certain jurisprudential theories, emphasizes the textual, discursive nature of the law, especially as articulated through judicial decisions [12,13]. This discursive understanding of law stresses the language used by courts to describe the cases before them and to articulate reasons for their decisions. We refer to these theories as the *discursive school*. Formal theorists focus on the rules announced by courts, while the discursive theorists are attentive to the metaphor, symbolism, narrative and normative rhetoric that courts use to describe and justify those rules.

In hierarchical judicial systems such as that of the United States, ‘apex courts’ serve as the final voice on the formal law. By dint of their position at the highest level of the hierarchy, their decisions are authoritative on the content and interpretation of legal rules. However, the *discursive* influence of apex courts is far less absolute; lower courts are formally bound by the decisions of higher courts, but they have no obligation to mimic their narrative voice or rhetorical style. There are many potential influences on the discourse in the lower courts, including how lawyers argue their cases and broader social and cultural forces. Nevertheless, their prestige and relatively high profile create obvious channels for apex courts to influence discourse in the lower courts. Whether and how they do so are empirical questions.

We define two potential mechanisms for the US Supreme Court to exercise discursive influence. The first is via authorship. Broadly speaking, the authorship mechanism functions via a mimetic process in which courts pick up and copy the discursive innovations of prior courts. This mechanism of influence is akin to that through which authors exercise influence within the literary sphere [14]. Any court can, in principle, exercise discursive influence through the authorship mechanism by dint of the fact that it can author and publish opinions.

The second influence mechanism we define is via selection, and it is unique to the Supreme Court. In the United States, the contemporary Supreme Court largely selects its own docket. Over time, the Court's mandatory jurisdiction has declined and in 1988, Congress adopted the Supreme Court Case Selections Act, which nearly eliminated the Court's mandatory jurisdiction [15]. Today the Court's docket is made up almost entirely of cases that it selects for itself. As a result, parties who have exhausted their appeals in the federal and state courts can petition for a hearing before the Supreme Court, but the Court is not obligated to hear them. Typically, the Court grants a few dozen of these *certiorari* petitions each year. The discretionary docket of the Court creates the second mechanism of discursive influence. Here, the Court selects a limited number of cases in the lower courts and publicizes them. Some of these cases have discursive innovations that the Supreme Court may copy itself, and future lower courts also repeat the innovations that were contained in the cases selected for attention by the Supreme Court.

**Figure 1.**
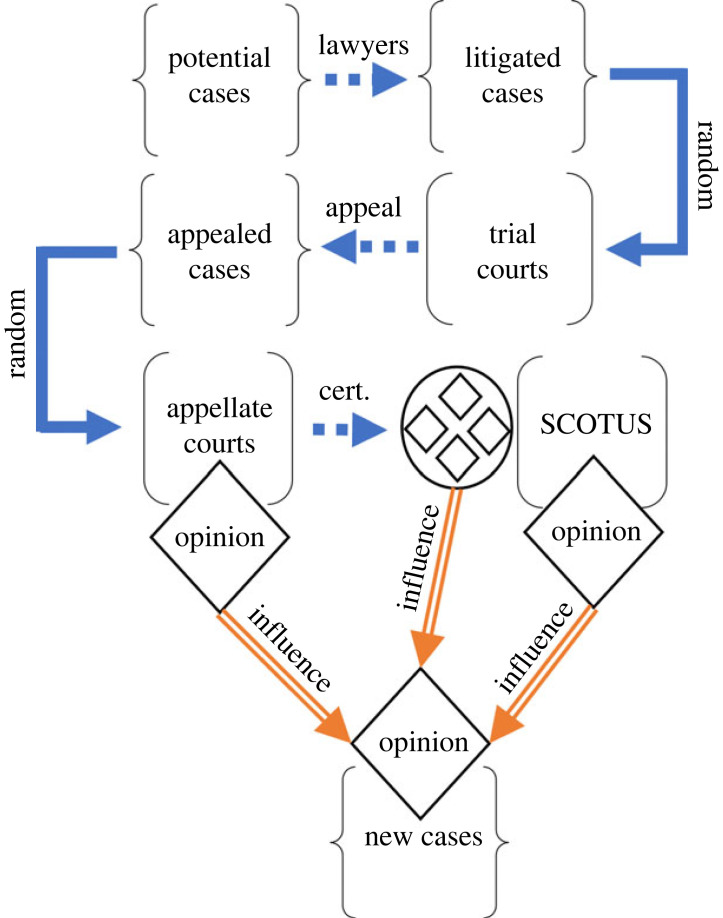
Illustration of the appeals process from the initial pool of potential cases through the Supreme Court, with the associated potential influence on future cases. Curly brackets denote pools of cases, straight brackets denote opinion-generating institutions. Dotted blue arrows indicate selection processes, while solid blue arrows indicate assignment processes. Rhombuses indicate decisions, and encircled rhombuses indicate case collections. Orange arrows indicate influence dynamics. (Online version in colour.)

Figure 1 provides an illustration of the relevant relationships that give rise to the judicial opinions of interest and, consequently, discursive influence.

The process starts with potential cases; these are the many disputes in society that could lead to litigation [16]. A first screening mechanism occurs when lawyers and clients decide which cases have merit and are worth proceeding with. The cases that move forward constitute the pool of litigated cases. These cases are randomly assigned to trial courts. At the trial court level parties file motions, trials are held and judgements are rendered. A second screening mechanism occurs after the decision of the trial court is entered, when parties decide whether to appeal or not; those cases that are appealed make up a second pool. A second random process assigns appealed cases to appellate court panels. At the appellate level, cases are briefed and argued, and judgements are entered. Appellate courts decide whether to issue opinions, and whether those opinions will be published. A third screening process occurs when the parties decide whether to petition for, and the Supreme Court decides whether to grant, *certiorari*. The *certiorari* decision creates a special pool of appellate cases—those that have been selected for review. The Supreme Court then sets its calendar, the parties brief their cases and make their oral arguments. The Justices then deliberate and make a decision, typically (but not always) issuing a judicial opinion. There are then subsequent cases that lead to new opinions. We are interested in three pathways for influence: from the pool of appellate opinions that are selected for review to new opinions (the selection mechanism); from Supreme Court opinions to new opinions (the authorship mechanism); and from appellate court opinions generally to new opinions.

### (b) Measuring discursive influence

We define discursive influence in judicial language as a meso-level phenomenon. At the micro-level are the legal rulings authoritatively announced by the Supreme Court, which lower courts are legally obligated to follow. An example of a legal ruling was the announcement of the Supreme Court in the case *Association for Molecular Pathology v. Myriad Genetics, Inc.*, 569 US 576 (2013), that human genes cannot be patented because DNA is a ‘product of nature’. A legal opinion could be made up of many different legal rulings, covering, for example, questions of procedure in addition to multiple substantive questions. At the macro-level is the subject matter of the opinion, which can be articulated at varying degrees of granularity. For example, the famous opinion *Miranda v. Arizona,* 384 US 436 (1966), addresses questions of criminal procedure and constitutional law, specifically the interpretation of the self-incrimination clause of the Fifth Amendment and the right to an attorney under the Sixth Amendment, and more specifically concerning police procedures in the course of interrogations. Again, judicial opinions can be, and often are, mixtures of different legal subject matter. The legal subject matter captures *what the opinion is about* and legal rulings are *what the court does*. For a legal theorist of the formal school, a description of a judicial opinion that extracted the micro- and macro-level features would be exhaustive.

The discourse in an opinion is neither the legal ruling nor the legal subject matter, but rather the explanatory, persuasive and justificatory language in an opinion. Legal discourse includes features related to writing style, argumentative structure and use of language in a judicial opinion. Legal theorists of the discursive school focus on this meso-level between the micro-level of legal rules and the macro-level of legal subject matter.

We can illustrate the point by defining a set of potential opinions {MG} all of which have the same subject matter as *Myriad Genetics*. These opinions would all concern the patentability of human genes. We can further define the set {MG} to also all have the same legal ruling: that human genes cannot be patented. Obviously, the actual decision, *Myriad Genetics* is an element of the set {MG}. Despite shared features at the macro- and micro-level, there could still be variation within the set {MG} in terms of, *inter alia*, the structure of the opinion, the justificatory language that is used and the narrative and writing style. The meso-level of discourse covers the variation within the set {MG}. Stated another way, Judge Posner has referred to the judicial writing style as ‘what is left out by paraphrase’ [17]. Every opinion within {MG} could be paraphrased in a similar, or identical, manner. The documents within {MG} could still differ in many respects; Judge Posner refers to those differences as stylistic; in this paper, we refer to them as meso-level, or discursive, characteristics.

Within the field of natural language processing, researchers have developed several different quantitative tools to estimate innovative language in large textual corpora. Some approaches seek to identify innovation at the level of concepts; for this task, identifying new terms or usages is especially important [18]. Alternatively, innovation can be understood as surprising combinations of words, where surprise could be estimated based on a predictive model [19]. For our investigation we apply a Document Influence Model (DIM) [5,6]. The DIM is well-suited to identify innovation at the level of discourse, the meso-level between macro-level subject matter and micro-level legal rules. The DIM identifies innovation in language within a subject matter category; innovation is not based on macro-level shifts in subject matter over time. The representation of texts within the model is also sufficiently coarse-grained that it will not capture nuanced differences in the combination of words that make up the micro-scale of legal rules.

The DIM is based on a *topic model,* a probabilistic model of document structure and generation built on the ‘bag-of-words’ representation of texts. Topic models decompose documents as distributions over a set of topics fixed at size *K*. Topics, meanwhile, are defined as distributions over a set of words in a fixed vocabulary of size *W*. Thus, the entire corpus is reduced to two sets of distributions: the document-topic distributions (denoted *Φ*) and the topic-word distributions (denoted *φ*). The topic proportion for topic *k* in document *j* is *Φ_kj_* and *φ_wk_* is the extent to which word *w* is associated with topic *k*. These values are calculated over an iterative expectation-maximization loop until convergence is reached [3]. The DIM builds on a topic model by allowing topics to change in content over time and by assigning document ‘influence scores’ (denoted *l*) that are higher for documents that predict how word frequencies change within a topic [6]. The influence scores calculated by the DIM track whether language usage in an opinion is *innovative*, meaning that it predicts future language use. This innovation occurs neither at the macro-level of subject matter (which would be captured in topic prevalence rather than topic content) nor at the micro-level of legal rulings (which are not captured at all by the topic model due to the coarse-graining that occurs through term-frequency vectorization and projection into the lower-dimensional topic space) but at the meso-level of discourse. The model calculates influence scores for each topic for each document (*l_kj_)*; the sum of topic influences weighted by topic proportions gives the total document influence score (*l_j_*).

The specific DIM we used is the *regression-based* Dynamic Influence Model (rDIM) [6], which differs from the standard DIM by allowing document-level covariates to influence topic-level influence. These could include variables such as author or publisher; in our model, we used the single covariate of circuit. Including this covariate allows the model to take into account the issuing court, in computing *l_kj_*; this approach facilitates the detection of the *selection* mechanism, as influence can be attributed to an opinion based in part on its court of authorship, not solely upon its text.

For our analysis, each observation is a written opinion, and the features that are extracted by the rDIM include the topic proportions *Φ_kj_* and the influence scores *l_kj_*. For posterior analysis, we retained several other document-specific metadata, including the year when the opinion was issued, the issuing circuit and the authoring judge. For Supreme Court cases, we labelled whether cases were taken up on *certiorari* jurisdiction or not. We also labelled whether appellate court cases were taken up for review.

We develop a novel approach to estimate the separate effects of the authorship and the selection mechanisms. We begin by running the rDIM on two separate, but related corpora. The first version, ORIGINAL, is a straightforward model run on both appellate court opinions and Supreme Court opinions. We then constructed an alternative dataset that we refer to as COPIED. In this run, we replace Supreme Court cases with a copy of the associated appellate court case. In the COPIED run, the Supreme Court is not an author; its only role is to repeat the opinions for the cases that it selected for review. The final step is a simple linear regression that predicts the document level influence score *l_j_* with the primary predictor of interest the issuing court (i.e. with the opinion as issued by the Supreme Court or an appellate court) and if the opinion was issued by the appellate court, whether it was associated with a case that was taken up for review. We include different sets of controls in alternative model specifications. We can compare the coefficients for the issuing court in both the ORIGINAL and the COPIED runs to distinguish between the influence derived from the selection mechanism compared with the influence derived from the authorship mechanism.

The data used for this study are published judicial opinions in the United States courts of appeals and US Supreme Court in the period 1970–2010 (inclusive). Data were accessed through the Caselaw Access Project hosted by Harvard Law School, which is available to the public at no cost. Our study period extends two decades before and two decades after the Supreme Court Case Selections Act of 1988, and one of the features included in our analysis is whether the Court was selected by *certiorari* or not. These data were gathered from the Supreme Court Database hosted at the University of Washington School of Law.

## 3. Results

Topic models are *supervised* in that the number of topics (*K*) usually is specified *a priori*. Our primary specification set *K* at 50, a number that balances the benefits of coarse-graining against the information loss of projecting term-frequency vectors into a lower-dimensional space. In keeping with best practices, we carry out our analysis with alternative values for *K* to test for robustness. These results are available in the electronic supplementary material.

**Figure 2.**
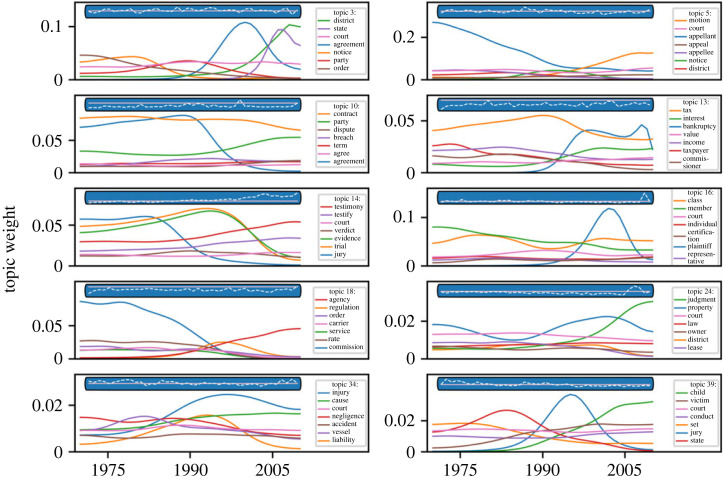
Changes in topic content and prevalence over time for an example set of topics in the *K* = 50 topic model. The coloured lines each represent the weight of a given word within a topic, which can grow or shrink over the study period (1970–2010). The dotted line in the blue bars represents the prevalence of the topic within the corpus over time relative to the average baseline (represented as a straight purple line).

Figure 2 illustrates the rDIM outputs for a selected set of example topics. Each topic has a prevalence in the corpus that can change over time; the prevalence of topic *k* in year *y* is the average value of topic prevalence for *k* for all opinions issued in year *y.* Figure 2 represents for a set of example topics, in each blue bar, the changes in topic prevalence compared with the baseline (1/*K*). In this corpus, topic prevalence remains relatively consistent over time. The rDIM also allows topic content to change over time. Every topic is a distribution over the vocabulary in a corpus; the content of a topic changes as the relative weights given to different words for that topic shift. The weights for every word in a topic can shift over time; figure 2 illustrates changes in the top six most highly weighted words in each of the topics.

For example, in topic 10, the most highly weighted words are associated with contracts; these include ‘contract’ as well as ‘breech’ and ‘term’. The prevalence of this topic is relatively stable, and somewhat below the average baseline, indicating that it is not as commonly discussed in the corpus as other topics. This is not surprising, given that contracts is primarily a state law subject area, and the corpus is of federal court opinions. The most substantial change in topic content is the decline in the word weight associated with the word ‘agreement’ over time. In topic 18, the most highly weighted words are associated with administrative law; these include ‘agency’, ‘regulation’ and ‘order’. Over the study period, the word ‘commission’ became less highly weighted, while the word ‘agency’ became more highly weighted. This change is in keeping with trends in US administrative law in which the most important locus of decision-making shifted from administrative bodies created during the New Deal period (often named commissions, such as the Securities and Exchange Commission) to administrative bodies created in the 1960–1970s period, which were often named, or referred to, as agencies (such as the Environmental Protection Agency).

**Figure 3.**
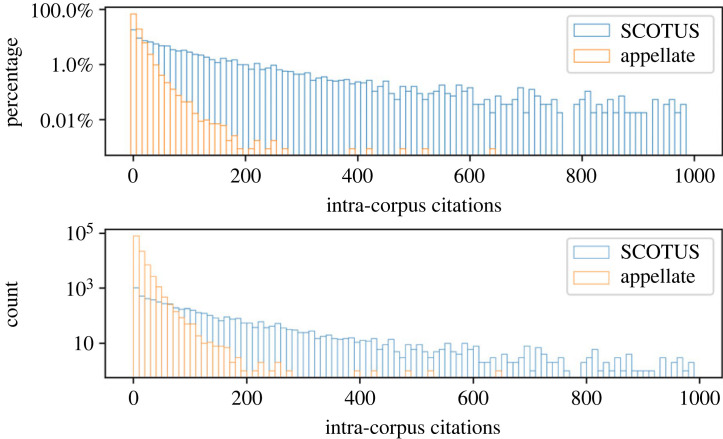
Citation histograms for Supreme Court and appellate court decisions. The top panel presents citation information as a percentage of the total number of issued opinions, and the bottom panel presents citation information as counts. Both scales are logarithmic.

Citation metrics are a common measure for influence within academic disciplines as well as for the judiciary [20,21]. The DIM was developed in part to address shortcomings in citation metrics [5,6], but citations may nonetheless be informative. Figure 3 provides citation information for the US Supreme Court as well as appellate courts. As is clear from figure 3, the Supreme Court is generally more highly cited, both in percentage terms (i.e. a higher percentage of Supreme Court cases are highly cited) and in absolute terms (the most highly cited cases are issued by the Supreme Court).

Citations in legal texts are used to clarify the authority for legal propositions within an opinion. They can be understood as tracking influence at the micro-level of legal rulings. As the apex court, the rulings that the Supreme Court issues are authoritative and binding across the entire judiciary. It is not surprising that it would have substantial influence at the micro-level of legal effects. In addition, the Supreme Court often uses its *certiorari* power to address issues of widespread importance, which create more opportunity for application, and therefore citation, than a typical appellate court decision.

**Figure 4.**
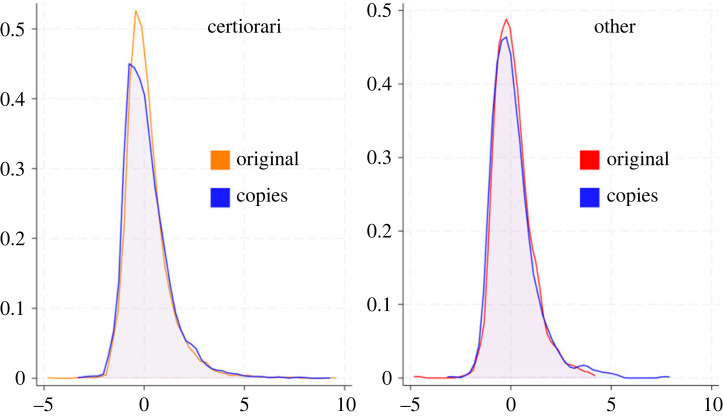
Smoothed histograms of influence scores for Supreme Court decisions in ORIGINAL and COPIED runs of the DIM. The left panel shows cases under the Court's *certiorari* jurisdiction; the right panel shows other cases. In the COPIED run, pseudo-Supreme Court cases are exact copies of the associated lower court opinion. The distributions largely overlap, but there are slightly higher influence scores in the ORIGINAL run for the *certiorari* cases.

Our primary method for comparing the selection and the authorship mechanisms for Supreme Court discursive influence is to compare a topic model run in which the original Supreme Court opinions are included (denoted: ORIGINAL) with a model run in which Supreme Court opinions are replaced with copies of the associated lower court opinion—i.e. the lower court opinion issued for the case that was taken up for review (denoted: COPIED). Figure 4 provides side-by-side histograms of influence scores for Supreme Court opinions in the two runs, with the left panel comprising cases under the Court's *certiorari* jurisdiction, and the right panel comprising other cases. Visually, the influence scores are extremely similar across the two groups.

We further explore this relationship through a series of regression analyses, which are reported in table 1.

**Table 1.** Regression analysis predicting influence scores based on issuing court, whether decision was selected for review and basis for Supreme Court jurisdiction. Standard errors in parentheses.

	(1)	(2)	(3)	(4)	(5)	(6)
	original	original	original	copied	copied	copied
SCOTUS	0.116***	0.140***		0.102***	0.082***	
*certiorari*	(0.016)	(0.016)		(0.017)	(0.014)	
SCOTUS	−0.061	−0.006		−0.132***	−0.068*	
*other*	(0.037)	(0.032)		(0.038)	(0.032)	
Selected	0.113***	0.133***	0.145***	0.208***	0.208***	0.207***
	(0.018)	(0.016)	(0.016)	(0.018)	(0.016)	(0.016)
R2	0.072	0.302	0.317	0.052	0.295	0.312
Topic		X	X		X	X
Judge			X			X
Obs	119 210	119 210	102 132	119 210	119 210	102 132

*p < 0.05, ***p < 0.001.

In each model, the outcome variable is the normalized total influence score (*l_j_* for observation *j*), and indicator variables are included for whether an opinion was issued by the Supreme Court after the case was selected through *certiorari* (*SCOTUS Certiorari*) or not (*SCOTUS Other*). Up to 1989 (the year after the Supreme Court Case Selections Act) 75% of cases issued by the Supreme Court are coded as *SCOTUS Certiorari*; from 1990 forward, 95% of Supreme Court cases are coded as *SCOTUS Certiorari*. For non-Supreme Court cases, we have a variable for whether the case was selected for review—i.e. whether *certiorari* was granted after the appellate court issued its decision (*Selected*). We account for a ‘burn in’ period in the rDIM (discussed in the electronic supplementary material) by excluding the first five years and final five years from our analysis. Linear and squared terms for year were also included in all models to account for potential time-correlated confounds.

Model 1 includes only these regressors, using the ORIGINAL dataset. In this model, *SCOTUS Certiorari* and *Selected* are both significant, with very similar coefficients, roughly 10% of a standard deviation increase in influence score. *SCOTUS Other* is not significant. Model 2 is the preferred specification and includes the variables for *Φ* for all topics as controls. The coefficient for *SCOTUS Certiorari* and *Selected* both increase somewhat between models 1 and 2; the coefficient for *SCOTUS Other* remains insignificant. Model 3 drops both types of SCOTUS cases and includes indicator variables for the authoring judge in addition to topic controls; the coefficient for *Selected* remains significant and roughly stable.

The ORIGINAL runs indicate that the selection mechanism is likely at work. Appellate court cases that are selected for review have significantly and meaningfully higher influence scores than those that are not selected for review, even after controlling for topic and the issuing judge. In addition, Supreme Court cases based on *certiorari* jurisdiction have significantly and meaningfully higher influence scores than other cases. There are, naturally, many unobserved confounds that interfere with robust causal analysis; it may be that there are variables that affect both the discursive influence of an opinion and the likelihood that the Supreme Court will take up a case for review. Such variables may not be adequately accounted for using year, topic and judge level controls. An ideal experiment would involve the Supreme Court's random selection of lower-level cases to test whether the act of selection alone is sufficient to increase influence scores. In the absence of such an experiment, the credence given to the selection mechanism must be discounted by the possibility that correlations between the selection-related variables and influence scores are driven by an unobserved additional variable.

The COPIED runs buttress the selection mechanism while indicating that the authorship mechanism plays some role as well. The most important comparison is between the preferred specifications, Model 2 (ORIGINAL) and Model 5 (COPIED). The coefficient for *SCOTUS Certiorari* in the COPIED run is significant and is somewhat over half the size of the coefficient in Model 2. This indicates that a decision that is a cut-and-paste copy of the lower court opinion, rather than the actual Supreme Court decision, is estimated to have at least a substantial portion of the influence as the actual Supreme Court decision. Nevertheless, the coefficient is smaller in the COPIED run, implying that the genuine Supreme Court decision does have some additional influence that can be attributed to authorship.

## 4. Discussion

Apex courts play important legal and social roles within polities around the world. At the very least, such courts serve as the ultimate arbiters of a host of legal questions and establish legal principles that are applied throughout a jurisdiction. But apex courts often are important cultural forces as well. In the United States, for example, the Supreme Court addresses the most highly controversial questions of the day, and its decisions can have profound economic and political consequences. Individual justices have even become pop cultural figures, garnering Hollywood biopics and attracting large followings of dedicated fans [22].

Beyond legal rulings and cultural impacts, apex courts may also exercise more subtle effects over the shape of legal discourse. The genre of the judicial opinion is an elaborate form of written communication that plays an important role in legitimizing the exercise of political power [23]. Although there are many differences between judicial opinions across legal cultures, they share the need to explain and justify decisions that often have profound consequences for affected parties. As famously articulated by Alexander Hamilton in Federalist 78, ‘The judiciary … has no influence over either the sword or the purse … it may be truly said to have neither FORCE nor WILL, but merely judgement.’ Given this tenuous position, the effective use of judicial opinions to ground the exercise of power by courts is essential to their long-term success. In their place atop judicial hierarchies, apex courts help determine how legal discourse is recruited in this legitimating project.

We have found that the US Supreme Court influences legal discourse—a meso-level phenomenon between the micro-level of legal rules and the macro-level of legal subject areas—both through its ability to select cases through review as well as (to a more limited extent) its own innovations. There is a robust literature that examines the relationship between the ‘core’ and ‘periphery’ in the cultural context [24], and whether innovation tends to occur within the well-connected, and often powerful centre, or at the edges of a social network, which may be less constrained and have more space for experimentation [19]. Our findings indicate that the Supreme Court—as the most core institution within the U.S. judiciary—plays an important role in influencing judicial discourse, but it does so not through its own innovation, but through its identification of innovation closer to the periphery. Future work could explore whether discursive influence tracks more fine-grained core-periphery variables. For example, a measure of discursive influence could be merged with a network representation of US Supreme Court cases, such as a citation or topic network, to estimate how the position of cases within the network affects that case's influence [25].

Our findings are also in keeping with prior work that indicates that the Supreme Court is becoming increasingly distinctive from the lower appellate courts in the United States [23]. That work also applied a topic model to appellate and Supreme Court cases and examined how topic prevalence within those corpora changed over time. It found that the distinctiveness of subject matter in Court opinions, as measured by topic prevalence, has increased over time. It further found that the subject matter in cases that the Court selected for review had not become more distinctive. These findings, along with the results discussed in this article, indicate that the Court may be developing a characteristic discursive style that may not be appropriate for, or attractive to, lower court judges.

Given the evolving role of the US Supreme Court, there would be value in additional work that examines its discursive influence in other historical periods. There are also other textual sources, such as treatises or law scholarship, that may exercise discursive influence; expanding the set of legal materials studied could also yield valuable insights. Another natural extension would examine the discursive influence of apex courts in other jurisdictions, and potentially cross-jurisdictional effects as well.

## Data Availability

The data are provided in the electronic supplementary material [26].
